# Chronic wounds alter the proteome profile in skin mucus of farmed gilthead seabream

**DOI:** 10.1186/s12864-017-4349-3

**Published:** 2017-12-02

**Authors:** Héctor Cordero, Monica F. Brinchmann, Alberto Cuesta, María A. Esteban

**Affiliations:** 1 Fish Innate Immune System Group, Department of Cell Biology and Histology, Faculty of Biology, Regional Campus of International Excellence “Campus Mare Nostrum”, University of Murcia, 30100 Murcia, Spain; 2 Department of Microbiology and Immunology, Rosalind Franklin University of Medicine and Science, North Chicago, IL 60064 USA; 3 Faculty of Biosciences and Aquaculture, Nord University, 8049 Bodø, Norway

**Keywords:** Proteome, Wounds, Skin mucus, Gilthead seabream (*Sparus aurata*), Teleosts, IgM

## Abstract

**Background:**

Skin and its mucus are known to be the first barrier of defence against any external stressors. In fish, skin wounds frequently appear as a result of intensive culture and also some diseases have skin ulcers as external clinical signs. However, there is no information about the changes produced by the wounds in the mucosae. In the present paper, we have studied the alterations in the proteome map of skin mucus of gilthead seabream during healing of experimentally produced chronic wounds by 2-DE followed by LC-MS/MS. The corresponding gene expression changes of some identified skin proteins were also investigated through qPCR.

**Results:**

Our study has successfully identified 21 differentially expressed proteins involved in immunity and stress processes as well as other metabolic and structural proteins and revealed, for the first time, that all are downregulated in the skin mucus of wounded seabream specimens. At transcript level, we found that four of nine markers (*ighm*, *gst3*, *actb* and *krt1*) were downregulated after causing the wounds while the rest of them remained unaltered in the wounded fish. Finally, ELISA analysis revealed that IgM levels were significantly lower in wounded fish compared to the control fish.

**Conclusions:**

Our study revealed a decreased-expression at protein and for some transcripts at mRNA levels in wounded fish, which could affect the functionality of these molecules, and therefore, delay the wound healing process and increase the susceptibility to any infection after wounds in the skin of gilthead seabream.

## Background

Teleost is the largest and most variable vertebrate taxon and most importantly, the earliest group of vertebrates possessing both an innate and adaptive immune system. Gilthead seabream (*Sparus aurata*; *Sparidae*; *Perciformes*; *Teleostei*) is a hermaphroditic protandrous marine species and one of the most farmed fish not only in Europe, but also worldwide with a global production of around 160,000 t in 2014 [1]. Intensive fish farming increases the occurrence of injuries and diseases, commonly associated with the appearance of wounds or ulcers in the skin, causing major economic losses [2, 3]. These injuries and diseases in the skin such as the white nodules from lymphocystis disease [4–6] or the physical wounds that increase the susceptibility of bacterial vibriosis [7] are devastating to farmed fish populations.

Skin mucus is mainly secreted by goblet cells in the skin of fish, protecting as a mechanical, physical, chemical, biological and immunological barrier against any external stressors [3, 8]. In recent years, skin mucus has become a hot topic as a faithful mirror of the immune status of fish [9]. Thus, many humoral immune activities such as proteases, antiproteases, peroxidases, esterases, alkaline phosphatase, lysozyme or immunoglobulins have been evaluated in skin mucus [10–12]. Apart from the individual characterization of antimicrobial peptides [13], immunoglobulins [14] or lectins [15], the recent advances in high throughput proteomics research methods have been used for identification and quantification of proteins [16]. Homology-driven proteomics is a major approach for identification of proteins in species where the sequences are not available [17]; however, identification of unknown proteins often relies on the similarity (rather than identity) when comparing with homologous protein sequences from phylogenetically related species [18], especially for the gilthead seabream, when the specific genome is not publically available and/or the transcriptome data are scarce.

Through this approach, the proteomic map of skin mucus has recently been studied in several fish species such as Atlantic cod [19], lumpsucker [20], European sea bass [21], and gilthead seabream [22, 23]. These studies have allowed the discovery of new molecules involved in protection and immunity of this mucosal surface. Besides changes in the skin mucus proteome, i.e. differentially expressed proteins have been studied after infection [24–27], from handling or crowding stress [28, 29], after parental care [30] and more recently after administration of different dietary supplements [29, 31]. However, despite the relevancy to fish health, there are no studies regarding the changes on the skin mucosae following injury so far.

The aim of this work was to study the alteration of the skin mucus proteome after inducing chronic wounds in gilthead seabream. This study was done using 2-DE followed by LC-MS/MS and provides a first idea about the changes of specific proteins involved in immunity, stress and metabolism, as well as structural proteins related to regeneration and healing processes present in skin mucus of gilthead seabream. Finally, we hypothesize that the proteomic levels in mucus and transcriptomic levels in skin are correlated as indicated by these markers as well as concentrations of IgM, which was the main systemic adaptive immune molecule found in skin mucus in our study.

## Results

**Fig. 1:**
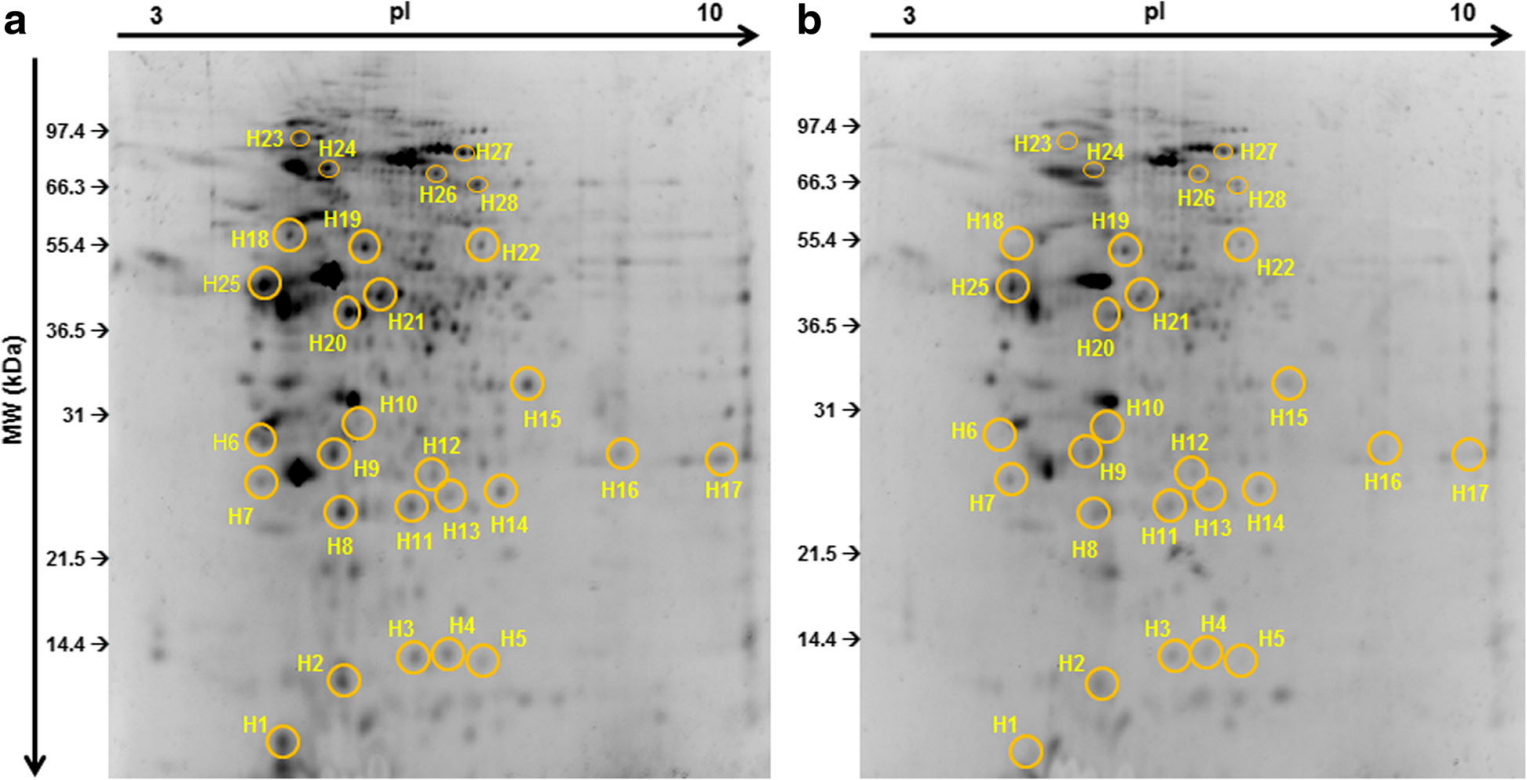
Representative 2-DE gels of skin mucus of control (**a**) and wounded (**b**) *S. aurata* specimens. Skin mucus proteins were isoelectrically focused on 17 cm IPG strips (*pI* 3–10) and subjected to 12.5% SDS-PAGE. The 2-DE gels were stained with SYPRO^®^ Ruby protein gel stain and the spots identified in (**a**–**b**) were annotated using the data from LC-MS/MS. The spot numbers represented in gels correspond to the protein identities mentioned in Table 2

The differential proteome of skin mucus of gilthead seabream after causing chronic wounds was studied through 2-DE (Fig. 1) followed by LC-MS/MS approach (Tables 1 and 2). The total differentially expressed proteins were clustered in four groups: immune-related (I), stress-related (II), structural (III) and metabolic (IV) proteins as described below.

**Table 1:** Details of the differentially expressed protein spots in skin mucus of *S. aurata* after chronic wounds

SN^a^	Protein name	Organism AN^b^	pI/MW^c^	S/C^d^	M_p_/U_p_^e^	Peptide sequence and e-value^f^
H1	Histone H4	*Oncorhynchus mykiss* P62797	11.4/11.4	76/19	2/2	**VFLENVIR** (2.9*10^−5^) **TVTAMDVVYALK** (0.002)
H2	Apolipoprotein A1	*Sparus aurata* AAT45246	5.3/15.9	151/39	3/3	**LLNLLSQAQTASGPMVEQASQDGR** (0.0068) **EYAETLQAKPEFQAFVK** (0.025) **VATALGEEASPLVDK** (0.016)
H3	Histone H2B	*Danio rerio* Q5BJA5	10.4/13.6	28/7	1/1	**LLLPGELAK** (0.0016)
H4	Cu/Zn Superoxide dismutase	*S. aurata* CAI79044	5.4/7.0	66/44	2/2	**HVGDLGNVTAGADNVAK** (4) **MLTLSGPLSIIGR** (0.14)
H5	Histone H2A	*D. rerio*	10.6/13.5	49/7	1/1	**AGLQFPVGR** (0.00014)
H6	14–3-3 protein beta/alpha-1	*O. mykiss* Q6UFZ9	4.6/27.7	143/15	2/5	**YLSEVASGDSK** (2.6*10^−8^) **YLSEVASGDSKK** (0.35) NLLSVAYK (8.3/10^−5^) VISSIEQK (1.3) DSTLIMQLLR (1.5*10^−5^)
H7	Apolipoprotein A1	*S. aurata* O42175	5.2/29.6	232/29	5/5	**AVLDVYLTQVK** (0.02) **AVNQLDDPQYAEFK** (0.0032) **IEEMYTQIK** (0.00025) **SSLAPQNEQLK** (0.00099) **TLLTPIYNDYK** (0.0014) **EVVQPYVQEYK** (0.092) **ITPLVEEIK** (0.0024)
H8	Phosphatidylethanolamine-binding protein 1	*S. aurata* FM145015	9.1/29.7	174/13	3/2	**LYDQLAGK** (28) **LYTLALTDPDAPSR (**0.0019) YGSVEIDELGK (0.00074)
H9	Apolipoprotein A1	*S. aurata* O42175	5.2/29.6	183/19	5/5	**IEEMYTQIK** (1.2) **SSLAPQNEQLK** (3.5) **TLLTPIYNDYK** (0.14) **EVVQPYVQEYK** (0.42) **ITPLVEEIK** (0.87)
H10	Actin cytoplasmic 1	*Ctenopharyngodon idella* P83751	5.3/42.1	53/7	3/3	**AGFAGDDAPR** (0.085) **DLTDYLMK** (0.089) **GYSFTTTAER** (6*10^−5^)
H11	Natural killer enhancing factor 2	*Larimichthys crocea* XP_010732927	5.9/21.8	278/26	6/2	**DYGVLKEDDGIAYR** (0.22) **EDDGIAYR** (21) IPLVADLTK (1.3*10^−5^) GLFVIDDK (0.41) QITINDLPVGR (0.00085) LVQAFQHTDK (0.34)
H12	ADP-ribosylation factor 3	*Takifugu rubripes* P61207	6.8/20.7	106/24	4/4	**ILMVGLDAAGK** (4*10^−7^) **MLAEDELR** (3*10^−5^) **DAVLLVFANK** (0.056) **QDLPNAMNAAEITDK** (0.17)
H13	Natural killer enhancing factor 1	*Osmerus mordax* ACO 09982	5.8/22.3	102/14	3/3	**LAPDFTAK** (26) **AVMPDGQFK** (18) **QITINDLPVGR** (0.0028)
H14	Glutathione S-transferase 3	*S. aurata* AFV39802	6.9/25.5	206/19	5/3	**FTGILGDFR** (0.00069) **MTEIPAVNR** (0.1) **TVMEVFDIK** (2.2) YLPVFEK (11) AILNYIAEK (0.79)
H15	Triosephosphate isomerase A	*S. aurata* FG266106	8.7/28.8	203/18	5/4	**IIYGGSVTGATCK** (0.3) **NVSEAVANSVR** (0.0059) **KNVSEAVANSVR** (1200) **GAFTGEISPAMIK** (4.9) FGVAAQNCYK (11)
H16	Triosephosphate isomerase B	*D. rerio* Q90XG0	6.5/27.1	76/12	3/3	**FFVGGNWK** (0.065) **GAFTGEISPAMIK** (5.7*10^−7^) **WVILGHSER** (0.037)
H17	Triosephosphate isomerase B	*D. rerio* Q90XG0	6.5/27.1	131/23	5/5	**FFVGGNWK** (6.1) **GAFTGEISPAMIK** (1.7*10^−6^) **WVILGHSER** (0.001) **HVFGESDELIGQK** (2.9*10^−6^) **VVLAYEPVWAIGTGK** (0.022)
H18	ATP synthase subunit beta	*Cyprinus carpio* Q9PTY0	5.1/55.3	317/28	10/10	**TIAMDGTEGLVR** (0.0043) **VLDTGAPIR** (1.8*10^−6^) **IPVGPETLGR** (7.4*10^−8^) **IMNVIGEPIDER** (1.1*10^−6^) **VVDLLAPYAK** (3*10^−5^) **IGLFGGAGVGK** (6.8*10^−6^) **TVLIMELINNVAK** (0.022) **VALVYGQMNEPPGAR** (5.4*10^−5^) **IPSAVGYQPTLATDMGTMQER** (0.0006) **AIAELGIYPAVDPLDSTSR** (0.0045)
H19	Actin-related protein	*T. rubripes* O73723	5.6/47.9	33/8	3/3	**FSYVCPDLVK** (0.062) **DYEEIGPSICR** (0.0066) **EVGIPPEQSLETAK** (0.14)
H20	Actin cytoplasmic 1	*Oreochromis mossambicus* P68143	5.3/42.1	144/15	5/5	**AGFAGDDAPR** (3.8*10^−8^) **VAPEEHPVLLTEAPLNPK** (0.0038) **DLTDYLMK** (0.024) **GYSFTTTAER** (1.5*10^−5^) **EITALAPSTMK** (0.066)
H21	Macrophage-capping protein	*L. crocea* XP_010735467	5.8/38.7	185/12	5/4	**TQVEILPQGK** (0.022) **MKTQVEILPQGK** (0.45) **MPELAESTPEEDSK** (0.16) **EIASLIR** (10) EGGVESGFR (1.8)
H22	Citrate synthase	*Katsuwonus pelamis* Q6S9V7	8.5/52.4	95/8	4/4	**DVLSDLIPK** (0.25) **ALGFPLERPK** (0.061) **VVPGYGHAVLR** (3.7*10^−5^) **IVPNVLLEQGK** (1.1*10^−6^)
H23	Heat shock cognate 71 kDa	*Oryzias latipes* Q9W6Y1	5.8/76.6	476/20	13/3	NQVAMNPTNTVFDAK (1.8*10 ^**−7**^ **)** SFYPEEVSSMVLTK (1.2*10 ^**−5**^ **)** **GQIHDIVLVGGSTR (0.0077)** VEIIANDQGNR (5.8*10^−6^) MKEIAEAYLGK (7.2*10^−5^) EIAEAYLGK (0.02) DAGTISGLNVLR (3.6*10^−5^) IINEPTAAAIAYGLDKK (1*10^−6^) STAGDTHLGGEDFDNR (0.0014) ARFEELNADLFR (5.5*10^−5^) FEELNADLFR (7.6*10^−7^) LLQDFFNGK (9.2*10^−6^) NGLESYAFNMK (0.00053)
H24	Heat shock cognate 71 kDa	*Ictalurus punctatus* P47773	5.2/71.6	306/14	8/2	TTPSYVAFTDSER (1.8*10 ^**−6**^ **)** **FELTGIPPAPR (0.00019)** VEIIANDQGNR (2.3*10^−7^) MKEIAEAYLGK (0.096) DAGTISGLNVLR (7.8*10^−7^) STAGDTHLGGEDFDNR (0.00012) FEELNADLFR (7.2*10^−8^) LLQDFFNGK (0.0026)
H25	Keratin type I	*O. mykiss* NP_001117848	5.2/51.9	521/14	9/0	KLEAANAELELK (1.7*10^−9^) LEAANAELELK (0.00012) LAADDFR (0.0068) TKYENELAMR (0.041) QSVEADIAGLKR (43) SDLEMQIEGLK (9.2*10^−5^) NHEEELLAMR (1.6) TRLEMEIAEYR (0.18) LEMEIAEYR (0.029)
H26	Complement component 3	*S. aurata* ADM13620	8.1/186.9	152/4	7/6	**TLYTPESTVLYR** (18) **DITYLILSR** (0.87) **VTGDPEATVGLVAVDK** (62) **SVPFIIIPMK** (13) **DSSLNDGIMR** (21) **VVPQGVLIK** (11) EYVLPSFEVK (100)
H27	Gelsolin	*S. aurata* HS984154	6.0/31.6	548/45	9/7	**QPGLQVWR** (0.035) **GGVASGFQHVVTNDMSAK** (13) **GDSFILDLGK** (0.059) **LHMVEEGEEPK** (25) **AFTEALGPK** (2.1) **TAIAPSTPDDEKADISNK** (0.00049) **GALYMISDASGTMK** (0.0044) **VSSVAPSSPFK** (0.0033) QAMLSPEECYILDNGVDK (1600) IENLDLKPVPK (54)
H28	Immunoglobulin M heavy chain	*S. aurata* AFN20639	6.1/51.2	50/2	1/1	**GFSPNSFQFK** (0.039)

^a^Spot number

^b^Accession number in NCBI or SwissProt databases

^c^Theoretical isoelectric point and molecular weight (kDa)

^d^Total score and coverage (%)

^e^Total matched peptides (Mp)/total unique peptides (Up)

^f^Unique peptides are in bold. Expect value (e-value) is noted for each peptide sequence

**Table 2:** List of proteins that are differentially expressed in skin mucus of *S. aurata* after chronic wounds

Spot	Protein name	Fold change	Previously detected in skin mucus?	References
H1	Histone H4 (H4)	↓ 0.01	Yes	[21, 23]
H2	Apolipoprotein A1 (APOA1)	↓ 0.04	Yes	[19–22, 29, 40]
H3	Histone H2B (H2B)	↓ 0.12	Yes	[20]
H4	Cu/Zn Superoxide dismutase (SOD)	↓ 0.09	Yes	[21–23]
H5	Histone H2A (H2A)	↓ 0.06	Yes	[23]
H6	14–3-3 protein beta/alpha 1	↓ 0.02	Yes	[19–23, 29]
H7	Apolipoprotein A1 (APOA1)	↓ 0.44	Yes	[19–22, 29]
H8	Phosphatidylethanolamine-binding protein 1 (PEBP1)	↓ 0.09	Yes	[22, 23]
H9	Apolipoprotein A1 (APOA1)	↓ 0.06	Yes	[19–22, 29, 40]
H10	Actin cytoplasmic 1 (ACTB)	↓ 0.32	Yes	[20–23, 29, 40]
H11	Natural killer enhancing factor 2 (NKEF2)	↓ 0.13	Yes	[21–23]
H12	ADP-ribosylation factor 3 (ARF3)	↓ 0.06	Yes	[29, 40]
H13	Natural killer enhancing factor 1 (NKEF1)	↓ 0.17	Yes	[20–23]
H14	Glutathione S-transferase 3 (GST3)	↓ 0.11	No	None
H15	Triosephosphate isomerase A (TPIA)	↓ 0.01	Yes	[19, 23, 29]
H16	Triosephosphate isomerase B (TPIB)	↓ 0.02	Yes	[19, 21, 23]
H17	Triosephosphate isomerase B (TPIB)	↓ 0.01	Yes	[19, 21, 23]
H18	ATP synthase subunit beta (ATB5B)	↓ 0.07	Yes	[20, 22, 23, 40]
H19	Actin-related protein (ARP)	↓ 0.46	Yes	[23, 24, 40]
H20	Actin cytoplasmic 1 (ACTB)	↓ 0.25	Yes	[19, 21–23, 40]
H21	Macrophage-capping protein (CAPG)	↓ 0.18	Yes	[22]
H22	Citrate synthase (CS)	↓ 0.09	Yes	[19]
H23	Heat shock cognate 71 kDa (HSC70)	↓ 0.12	Yes	[20, 22, 23, 40]
H24	Heat shock cognate 71 kDa (HSC70)	↓ 0.27	Yes	[20, 22, 23, 40]
H25	Keratin type I (KRT1)	↓ 0.11	Yes	[19–23, 29]
H26	Complement component 3 (C3)	↓ 0.36	Yes	[21, 29, 40]
H27	Gelsolin (GSN)	↓ 0.41	Yes	[21, 22, 40]
H28	Immunoglobulin M heavy chain (IgM)	↓ 0.07	No	None

↓ indicates under-expression of the proteins at *p* < 0.01. In addition, a literature-based comparison about presence of these proteins in skin mucus of other fish species after 2-DE spot detection is included

### Immune-related molecules

The differential skin mucus proteome of gilthead seabream showed a general decrease of some proteins involved in several immune routes (Tables 1 and 2). One of the most important components of both innate and adaptive immunity, the complement molecule C3 (spot H26), was identified and down-regulated after chronic wounding. Similarly, APOA1 was identified in different parts of the gels (spots H2, H7 and H9) and also showed down-regulated expression in all the analysed protein spots.

It is well-known that some histones may act as antimicrobial peptides [32]. We have identified H2A (spot H5), H2B (spot H3) and H4 (spot H1) to be differentially down-regulated in the skin mucus from wounds of gilthead seabream. Finally, the main component of the adaptive immunity, IgM (spot 28), identified for the first time in skin mucus after 2-DE methodology, showed an interesting down-regulation after chronic wounding in skin mucus compared to control fish.

### Stress-related molecules

Chronic wounds in the skin also altered some stress-related proteins in the mucus of gilthead seabream (Tables 1 and 2). Peroxiredoxins are a family of antioxidant enzymes that protect cells from oxidative damage [33]. Some of the most studied peroxiredoxins, identified here such as NKEF1 (spot 13) and NKEF2 (spot 11), were down-regulated after chronic wounding in skin mucus of gilthead seabream. Furthermore, we have identified SOD (spot H4), GST3 (spot 14) and HSC70 (it was identified in two parts of the proteome map, spots H23 and H24), which were also down-regulated after chronic wounding (Table 3).

**Table 3:** Information of primers used for qPCR study

Gene names	Accession number	Amplicon size	Sequence (5′ → 3′)
Immunoglobulin mu heavy chain	JQ811851	113	F: CAACATGCCCAATTGATGAG R: GGCACGACACTCTAGCTTCC
Complement component 3	HM543456	106	F: CGCTCTTCTTGCTCTGGTGA R: CTGAGTTGATCCGTAGCCCC
Histone 2b	AM953480	174	F: AGACGGTCAAAGCACCAAAG R: AGTTCATGATGCCCATAGCC
Heat shock cognate 71 kDa	HS987272	124	F: GCCATGAACCCAACCAACAC R: GGCGGGTGTTGTCATTGATG
Superoxide dismutase	AJ937872	103	F: TCACGGACAAGATGCTCACT R: TCCTCGTTGCCTCCTTTTCC
Glutathione s-transferase	JQ308828	111	F: AGCGCTACCTTCCAGTGTTC R: CCTCCAACATCAGGGTGCAT
Gelsolin	HS984154	105	F: GCCATCAGAGCAACAGAGGT R: CTCACTGCCACACCACTGAT
Actin beta	AF316854	352	F: GGCACCACACCTTCTACAATG R: GTGGTGGTGAAGCTGTAGCC
Keratin 1	FJ744592	105	F: AGAGATCAATGACCTGCGGC R: CCCTCTGTGTCTGCCAATGT
Elongation factor 1 alpha	AF184170	115	F: TGTCATCAAGGCTGTTGAGC R: GCACACTTCTTGTTGCTGGA
Ribosomal protein s18	AM490061	109	F: CGAAAGCATTTGCCAAGAAT R: AGTTGGCACCGTTTATGGTC

### Structural molecules

Our study indicated that structural proteins also play a major role in chronic injury of skin. We have identified ACTB (spots H10 and H20), ARP (spot 19), CAPG (spot H21), KRT1 (spot H25) and GSN (spot H27), and shown down-regulation in all cases with the lowest levels in KRT1 (Tables 1 and 2).

### Metabolism molecules

Important proteins involved in several metabolic routes were identified in the present study. We found differential expression of YWHAZ (spot H6), PEBP1 (spot H8), ARF3 (spot H12), TPIA (spot H15), TPIB (spot H16 and H17), ATPB5B (H18) and CS (H22). All of these were down-regulated after chronic wounds in skin mucus of gilthead seabream (Tables 1 and 2).

### Functional level of IgM

**Fig. 2:**
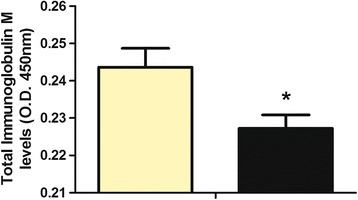
Total IgM levels detected by ELISA in skin mucus of control (yellow bar) and wounded (black bar) *S. aurata* specimens. Results are expressed as mean ± SEM (*n* = 3). The asterisks indicate significant differences (when *p* < 0.05) between control and wounded groups

Our ELISA study with specific antibodies for total IgM of gilthead seabream showed a significant decrease of total IgM levels detected in skin mucus after chronic wounds compared to the levels detected in the skin mucus of control group (Fig. 2).

### Transcript levels

**Fig. 3:**
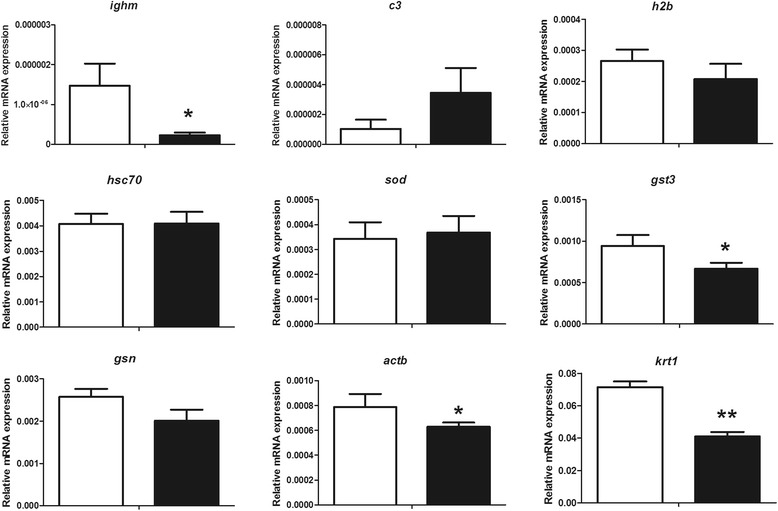
Expression levels of some immune-related genes such as immunoglobulin *mu* heavy chain (*ighm*), complement component 3 (*c3*), histone 2b (*h2b*); some stress-related genes such as heat shock cognate 71 kDa protein (*hsc70*), superoxide dismutase (*sod*), glutathione s-transferase 3 (*gst3*); and some structural-related genes such as gelsolin (*gsn*), actin beta (*actb*), keratin 1 (*krt1*) in the skin of control (white bars) or wounded (black bars) *S. aurata* specimens. Transcripts were quantified by qPCR and normalised using the geometric average of the reference genes elongation factor 1 alpha (*ef1a*) and ribosomal protein S18 (*rps18*). The values are presented as mean ± SEM (*n* = 6). The asterisks indicate significant differences (*p < 0.05 or **p < 0.01) between control and wounded groups

Due to the importance of the skin mucus markers in the processes of immunity, inflammation, stress, skin regeneration and wound healing, we have selected and studied the gene expression profile of several immune-related (*ighm*, *c3* and *h2b*), stress-related (*hsc70*, *sod* and *gst3*) and finally structural-related molecules (*gsn*, *actb* and *krt1*) (Fig. 3). Regarding immune-related genes, *ighm* was significantly down-regulated in the wounded group, while the increase and decrease observed in *c3* and *h2b*, respectively, were not significant compared to the control group. Little variations were observed at transcript level in the case of stress-related genes, where only *gst3* showed a significant down-regulation in the wounded group, while *hsc70* and *sod* remained unaltered compared to the control group. Finally, the structural genes were the most affected by chronic wounds, as all of them the trend were down-regulation, with significant changes in the case of *actb* and *krt1*, the latter being the most affected molecule at transcript level in the wounded group compared to the control groups.

## Discussion

Many factors such as stress by temperature, hypoxia, transportation, crowding, seasonal or dietary changes, can affect directly the skin integrity in farmed fish. Most of the available studies have tried to improve the skin healing by dietary supplementation of diets with vitamin C [34], β-Glucans [35, 36] and minerals with different combinations of vitamins and glucans [37]. But curiously, the global molecular changes produced by wounds have scarcely been studied in fish. Only the transcriptomic changes using microarray technology in the skin after skin and scale regeneration was reported [38]. The present study represents the first proteomic approach in the study of fish skin wounds.

From our own studies on fish skin mucus [11, 12, 39] and with proteomic tools [19, 26, 29], we provide evidence that 2-DE followed by LC-MS/MS provides good resolution and high performance for protein detection. One of the limitations of this approach could be the limited range of molecular weights available, thus mucins and other high molecular weight proteins have been undetected in these works. A recently published proteome map of gilthead seabream with more than 2000 proteins used 1-DE gels and mass spectrometry and any mucin was identified [40].

In the present study both protein levels and transcript levels were studied. In general one must have transcripts to make proteins, however due to, among others, RNA turnover rate, RNA localisation and protein turnover rate the changes in protein amount and RNA amount do not need to be the same. We found that *ighm*, *gst3*, *actb*, *krt1* transcripts were changed, whilst other transcripts were not significantly changed even if changes in proteins were observed.

Skin mucus is the first barrier of defense in fish, which contains immune components involved in both innate and adaptive immunity. In the present study we have demonstrated the presence of C3, APOA1, H2A, H2B, H4 and IgM. C3 can, upon cleavage, act as a chemoattractant (recruit immune cells), as opsonin (coat pathogens) to increase phagocytosis or as an agglutinin (coagulate pathogens) [21]. C3 was previously found in skin mucus of European sea bass [21]. While in the present study C3 was under-expressed in chronic wound specimens, in another study C3 was over-expressed after crowding stress in skin mucus of gilthead seabream [29]. At transcriptional level, no changes in *c3* expression are reported in the skin of gilthead seabream after chronic wounds. Accordingly, in our previous study the transcript levels of *c3* were also unaltered in skin after crowding stress despite the protein differential expression in skin mucus of gilthead seabream [29].

APOA1 is the major component of high density lipoprotein in serum [41], which also act as a negative acute phase protein [42], and possesses bactericidal activity in vitro [43]; however, despite the previous finding of APOA1 as a conserved marker in skin mucus of European sea bass [21], Atlantic salmon [27], lumpsucker [20], Atlantic cod [19, 26] and gilthead seabream [29], its role in mucus is still unknown. Our study suggests that it plays a role as a negative acute phase protein may also occur in skin mucus as we found that APOA1 was under-expressed after chronic injury.

In addition to their classical role as histones folding DNA into chromatin, H2A, H2B and H4 are also known as antimicrobial peptides [32, 44], a role especially notable for H2A and H2B in skin mucus of fish [45, 46]. The histone H4 deserves more attention since previous studies have found this histone in the skin mucus [21], but little is known about its role as antimicrobial peptide. The under-expression of these three histones in skin mucus after chronic wounds may facilitate the entry of potential pathogens resulting in loss of immune defense. However, in sharp contrast with other studies where *h2b* was mostly up-regulated after virus and/or bacterial infections [44], in our study, *h2b* showed no differences at transcript level between control and wounded groups.

The main effector of the humoral systemic adaptive immunity, IgM, has been widely studied by ELISA in skin mucus of fish maintained under many different conditions and in several fish species [11, 12, 39]. However, in the present study we have identified IgM in a fish skin mucus proteome using 2-DE technology for the first time. IgM was under-expressed in skin mucus after chronic wounds. At transcript level, the down-regulation of *ighm* demonstrated the key role of this immunoglobulin in this type of stress. In many cases, the down-regulation of one gene or even the protein level are not correlated with the activity, but importantly in our study the IgM levels were also decreased when specific antibody was used. Further studies on this topic will help to characterize and elucidate the IgM functions in skin mucus as adaptive immunity players.

Here we hypothesize that the lower levels of these immunological proteins could promote the entry of pathogens into the fish body since the epidermis was removed and the skin was, therefore, interrupted. However, these lower levels could be related to the fact that abrasion promotes overproduction of immature mucus high in mucins unmeasurable in 2D gel analyses, which could lead to underestimation of the detected proteins. In this context, the knowledge on the production of mucins during the wound healing process would be essential.

On the other hand, it has been previously reported an increased inflammatory response ie. changed cytokine expression profile in wound healing on day 14 after wounding [36]. By contrast, in the present paper, we have not detected any cytokine, which does not necessarily mean absence of inflammation, but cytokines could be undetectable in our study because of their low molecular sizes and/or their limited presence in skin mucus. The differences in results could also be because the inflammatory response was detected mainly after 14 days [36], whilst our results were from 5 days of wound healing. There is a close relation between stress and immunity, especially in lower vertebrates such as fish, in which, for instance, cytokines and neuropeptides are performing roles in both neuroendocrine and immune system [47]. Another example of this relationship between stress and immunity are peroxiredoxins, which may act as modulators of inflammation in pathogen infection and in protection against cell death, tissue repair after damage, and tumour progression [48]. According to our results, in which NKEF1 and NKEF2 are under-expressed in skin mucus after chronic wounds, fish NKEFs expression, at either gene or protein level, is regulated by LPS treatment and pathogens including bacteria, viruses and parasites [33]. Concretely, NKEFs have been previously found in skin mucus of gilthead seabream [23], and over-expressed after crowding stress [29]. Our results indicate the opposite expression regulation when fish were stressed by crowding or damage and chronic wounds.

Also in close relation with the immunity, SOD is an enzyme that protects the tissue against oxidative stress by regulating various reactive oxygen (ROS) and reactive nitrogen species molecules [49]. In addition, T cell activation induces the secretion of SOD [50]. SOD was also identified previously in skin mucus of gilthead seabream [22, 23], however, this is the first time that this protein was demonstrated to be differentially expressed in skin mucus, but curiously no changes were found at transcript levels of *sod* in the skin after causing the wounds. In sharp contrast with our data, *sod* was up-regulated after in vitro exposure with different metals in gilthead seabream erythrocytes [51] as well as in gilthead seabream SAF-1 cell line [52].

GSTs are the superfamily of phase II detoxification enzymes that play crucial roles in cellular defense [21]. Some members of this superfamily have been previously identified in skin mucus of fish [9], reducing the amount of proteins in Atlantic cod after *V. anguillarum* infection [26] or increasing the amount of protein in gilthead seabream after probiotic intake [29]. In the present study GST3 was identified for first time in skin mucus, and was under-expressed after chronic wounds. At the transcriptional level, *gst3* was the only stress marker which was significantly down-regulated in skin of gilthead seabream after chronic wounds. By contrast, a previous study also in gilthead seabream reported an up-regulation of *gst3* in the liver after nanoparticle exposure [53]. However, there is no further information is available on the effects of *gst3* in the skin of teleost fish.

HSPs are part of a superfamily of stress proteins, highly conserved across species, often classified based on their molecular weight [21]. Both HSP70 and HSC70 may have similar cellular roles and have been previously found in skin mucus [20–23]. HSC70 can be mildly modulated by stressors such as heat [54], pathogens [55], and heavy metals [56]. According to these previous studies, at protein level, the present study demonstrated the under-expression of HSC70 in skin mucus after chronic wounds. By contrast, at transcript level, *hsc70* remains unaltered after wounding.

Some metabolic proteins have also been found to be under-expressed in skin mucus after chronic injury. PEBP1 was found in the mapping of gilthead seabream skin mucus [22], similar to YWHAZ [22, 23]. Moreover, YWHAZ was found in skin mucus of other fish species such as Atlantic cod [19], lumpsucker [20] and Atlantic salmon [24]. In agreement with the present study, it was reported that YWHAZ, ARF and TPIA were under-expressed after crowding stress in skin mucus of gilthead seabream [29]. CS and ATP5B were previously found in the skin mucus of Atlantic cod [19] and gilthead seabream [23], but this is the first time that these proteins were found differentially expressed in skin mucus of fish.

Beta actin (ACTB) is a multifunctional protein involved in cell motility and phagocytosis. It has been reported that ACTB can be fragmented after stress [27]. This fact could explain the under-expression of ACTB found in our study. In agreement with this result, ATCB was also under-expressed after crowding stress [29]. At transcript level, *actb* was also down-regulated after chronic wounds in gilthead seabream. The variations of *actb* in both skin and skin mucus in the present and other studies demonstrate that this molecule is highly influenced by the different stimuli, and therefore, its use as reference gene should be avoided, or at least reconsidered, in this tissue and fish species. In close relation with ACTB, ARP, CAPG and GSN were previously found in skin mucus of gilthead seabream [22, 23], however little is known about the interaction of all these proteins in stress processes since this is the first time that ARP and CAPG were found differentially expressed in skin mucus of fish. On the other hand, GSN was also expressed in skin mucus of gilthead seabream after stress stimuli [40]. The transcript levels of *gsn* were studied in gilthead seabream for first time in the present article, reporting no changes in the expression of *gsn* in the skin of gilthead seabream after chronic wounds. The importance of *gsn* in the skin remains unknown since most of the studies were focused in the corneal development and embryogenesis of zebrafish [57, 58].

KRTs are heteropolymeric intermediate filaments containing type I (KRT1) and type II (KRT2) keratins. These molecules have been reported in skin mucus of many fish species [9]. In the present study KRT1 was under-expressed after chronic wound in a similar fashion than KRT2 was under-expressed in skin mucus after infection [26]. In contrast, KRT1 was over-expressed in skin mucus after crowding stress [29]. It has been reported that KRTs play a role in the regulation stress-resistance in epithelial cells [59]. In addition, KRTs have been associated with pore-formation activities in skin mucus of fish [60]. A recent article reported an overexpression of KTR2 in skin mucus after different chronic stressors such as shaking, sounds and light flashes [40]. At transcript level, the present study revealed a great down-regulation of *krt1* after chronic wounding. Despite of the diversity of keratins reported in fish [61], there is very little information about the changes produced by these molecules at transcript levels in fish. Overall, it seems that KRTs are essential to maintain the proper function of skin mucus. The present findings of KRT1/*krt1* at both protein and transcript levels suggest an important role of this molecule after chronic wounds in the skin mucosae that it deserves to be studied in depth.

## Conclusion

This study shows for first time the fish skin mucus proteome map of wounds. Thus, chronic wounding leads to a down-regulation in skin mucus proteins which are immune-related (C3, APOA1, H2A, H2B, H4 and IGM) and stress-related (NKEF1, NKEF2, SOD, GST3 and HSC-70), but also molecules involved in metabolism (PEBP1, YWHAZ, TPIA, TPIB, ARF, CS and ATP5B) and structural proteins (ATCB, ARP, CAPG, GSN and KRT1). The chronic wounding also leads a down-regulation of the transcripts corresponding to four of these proteins found in the skin of wounded specimens. These early alterations after chronic wounds could increase the susceptibility to pathogen infection due to the decrease in immune-related proteins as immune barrier and because of the decrease in structural proteins of the physical barrier, allowing for penetration of pathogens and, therefore, increasing the vulnerability of the fish.

## Methods

### Animal care

Forty specimens of gilthead seabream (*S. aurata*) (4.7 ± 1.3 g and 7.4 ± 0.6 cm), obtained from a local farm (Murcia, Spain), were kept in running seawater aquaria of 250 L (water flow 900 l h^−1^) at 28 ‰ salinity, 22 °C and a photoperiod of 12 h light: 12 h dark. Fish were fed daily at 2% rate of fish biomass per day with commercial diet (Skretting). All the fish handling procedures were approved by the Ethical Committee of the University of Murcia (Permit Number: A13150104).

### Chronic wounds

**Fig. 4:**
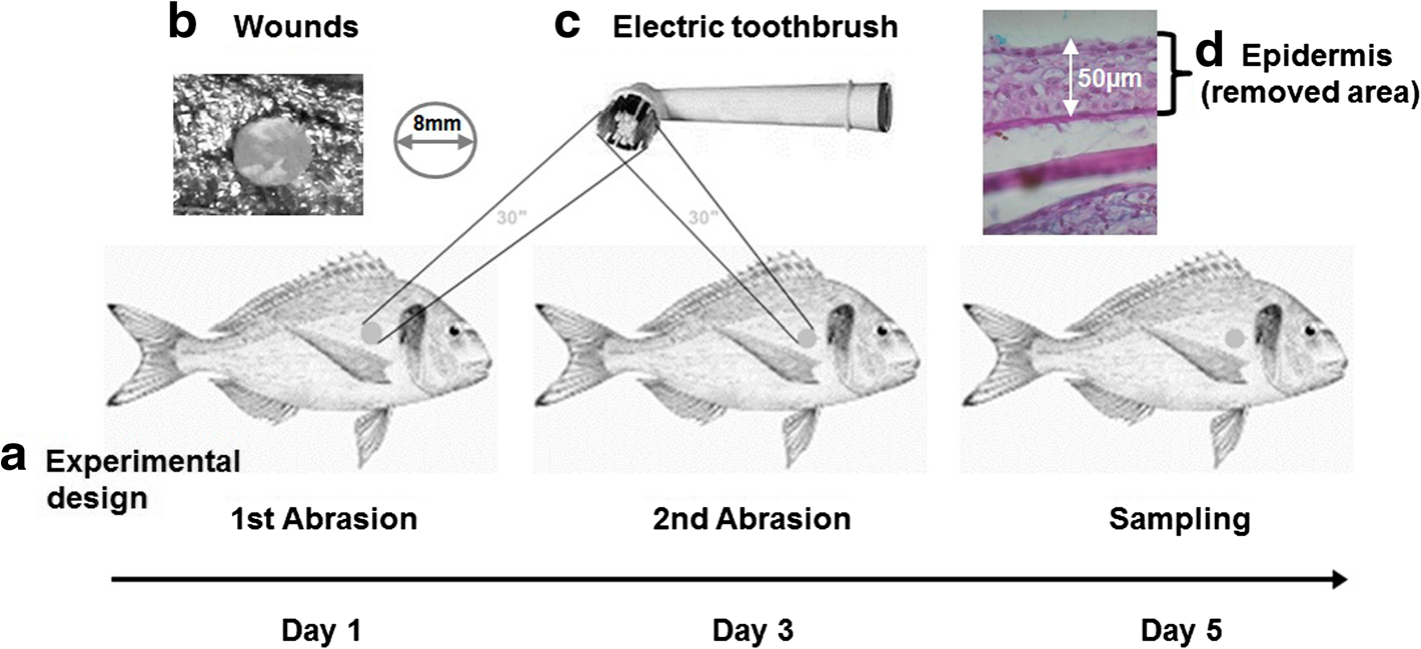
Illustration of the wounding model procedure on the skin of gilthead seabream (*S. aurata*) (**a**). Fish were wounded with an electric toothbrush for 30 s on both body sides to remove around 50 μm depth of epidermis in wounds of 8 mm of diameter (**b**, **c**, and **d**). The image of fish model was used after permission from the Food and Agriculture Organization (http://www.fao.org/fishery/culturedspecies/Sparus_aurata/en)

Fish were anesthetized with 100 mg L^−1^ of MS-222 (tricaine methanesulfonate; Sigma-Aldrich). Chronic wounds with a diameter of 8 mm and around 50 μm of depth were induced in the skin with an electric toothbrush (PRIMO) used for 30 s in each body side of the fish specimens (Fig. 4). The procedure was repeated twice each two days and sampled two days after the last abrasion (Fig. 4). The control group was handled in a similar manner as control fish without triggering wounds.

### Mucus and tissues samples

Twenty fish per group were anesthetized as described above prior to sampling. Mucus was gently scraped off from the skin surface, avoiding blood, urine and faeces during collection [62]. In order to obtain a large enough amount of mucus, mucus samples from 10 fish were pooled as described elsewhere [29] resulting in two pools/groups. Mucus was transferred into tubes of 15 ml and stored at −80 °C until use. Skin tissue was collected in QIAzol lysis reagent (Qiagen) and stored at −80 °C for subsequent RNA extraction.

### Histological analysis

Skin samples (*n* = 6) were collected and processed as described elsewhere [63]. Skin samples were sectioned at 5 μm and stained with periodic acid–Schiff (PAS; Merck) according to the manufacturer’s instructions. Images were obtained under a light microscope (Leica DM6000B) with a digital camera (Leica DFC280) and processed by Leica Application Suite V 2.5.0. Software.

### Mucus protein purification

Each sample was solubilised with 1 mM DTT and 1.5 mM EDTA, which serves to act as a mild mucolytic agent [64]. Next, after two rounds of sonication for 6 s followed by cooling for 1 min, samples were centrifuged at 20,000 g for 30 min at 4 °C. The supernatant containing the soluble mucus proteins was desalted with proteomic grade water (G Biosciences) using centrifugal filters of 3 KDa (VWR) by spinning 3 times at 14,000 g at 4 °C with 0.2 ml of ice cold water each time. The dialysed protein solution was further purified by 2D clean-up kit (Bio-Rad) following the manufacturer’s instructions.

### 2-DE

The samples obtained after the 2D clean-up were resuspended in 2D lysis buffer (Bio-Rad) containing 7 M urea, 2 M thiourea, 1% (*w*/*v*) ASB-14, 40 mM Tris base, 0.001% bromophenol blue and 50 mM DTT (Sigma-Aldrich) and 0.5% (*v*/v) Biolytes 3–10 ampholyte (Bio-Rad). The protein content of the solubilised samples was estimated using Qubit protein assay (Life Technologies). Two hundred μg of proteins for each sample were rehydrated in 17 cm 3–10 IPG strips (Bio-Rad) and isoelectric focusing (IEF) was carried out using protean IEF cell (Bio-Rad). After IEF, the electro-focused IPG strips were reduced and alkylated for 15 min each in equilibration buffer containing 6 M urea (Sigma-Aldrich), 0.375 M Tris-HCl pH 8.8 (Bio-Rad), 2% (w/v) SDS (Sigma-Aldrich), 20% (v/v) glycerol (Merck) with 0.2% (w/v) DTT (Sigma-Aldrich) or 0.3% (w/v) iodoacetamide (Bio-Rad), respectively. The equilibrated strips were loaded on 12.5% polyacrylamide gels to perform SDS-PAGE [65], run on PROTEAN II system (Bio-Rad). The gels were stained overnight with SYPRO^®^ Ruby Protein Gel Stain (Life Technologies) according to the supplier’s protocol. Gel image documentation was carried out using ChemiDocTM XRS imaging system (Bio-Rad). Raw pictures were analysed using PDQuest Advanced software version 8.0.1 (Bio-Rad) including detection of spots, normalization using local regression, spot matching and differential expression analysis. Protein spots were considered as differentially expressed when expression level was at least 1.5-fold different compared to the control group and when the differences were detected as significant at *p* < 0.01 by two tailed Student’s t-test.

### LC-MS/MS analysis

Spots from SYPRO-stained gilthead seabream skin mucus 2-DE gels (*n* = 6) were picked, excised and subjected to in-gel reduction, alkylation, and tryptic digestion using 2–10 ng/μl trypsin (V511A; Promega) as described elsewhere [66]. Peptide mixtures containing 0.1% formic acid were loaded onto a nanoACQUITY UltraPerformance LC (Waters), containing a 5 μm Symmetry C18 Trap column (180 μm × 20 mm; Waters) in front of a 1.7 μm BEH130 C18 analytical column (100 μm × 100 mm; Waters). Peptides were separated with a gradient of 5–95% acetonitrile, 0.1% formic acid, with a flow of 0.4 μl min^−1^ eluted to a Q-TOF Ultima mass spectrometer (Micromass/Waters). The samples were run in data dependent tandem mass spectrophotometry (MC/MC) mode. Peak lists were generated from MS/MS by Mascot Distiller Workstation and submitted to MASCOT search engine (version 2.5.1) and searched against NCBInr with the following parameters: maximum one missed cleavage by trypsin, peptide mass tolerance 100 ppm, MS/MS ion tolerance set to 0.1 Da, carbamidomethylation of cysteine selected as fixed modification and methionine oxidation as variable modification. Protein hits not satisfying a significance threshold (*p* < 0.05) or with low sequence coverage were further searched against Swissprot and vertebrate EST (expressed sequence tags) databases, taxonomy *Actinopterygii*.

### Primer design

Primers were designed by OligoPerfect™ Designer (Life Technologies) from *S. aurata* sequences that are available in NCBInr database. Details regarding oligonucleotide primers and their attributes are given in Table 3.

### Gene expression analysis

The mRNA levels corresponding to nine differentially expressed immune-related, stress-related and structural proteins in the skin of the experimental fish were analysed by real-time PCR (qPCR). RNA was extracted individually from 50 mg of skin from six specimens of gilthead seabream from both ulcered and control groups using QIAzol lysis reagent method (Qiagen) as described elsewhere [67]. The quality of total RNA was checked on a 1.2% agarose gel, followed by quantification using the Qubit^®^ RNA assay kit and Qubit^®^ 2.0 fluorometer (Life Technologies). The complementary DNA (cDNA) was synthetised from 1 μg of RNA using QuantiTec Reverse Transcription Kit (Qiagen). Ten times diluted cDNA was used to conduct qPCR on a ABI PRISM 7500 instrument (Applied Biosystems) as described elsewhere [21], using SYBR Green PCR Core Reagents (Applied Biosystems) and the 2^−ΔΔCt^ method [68]. Each plate subjected to qPCR contained a negative control for cDNA template (water) as well as a control for reverse transcription. No amplification product was observed in negative controls and neither primer-dimer formation nor secondary structures were observed in any case. All qPCR reactions were carried out in duplicate and quantification cycle (Ct) values of each gene (target) were converted into relative quantities. Normalization factors were calculated as the geometric mean of relative quantities of reference genes elongation factor 1 alpha (*ef1a*) and ribosomal protein S18 (*rps18*) using the BestKeeper^©^ algorithm [69], which have been previously reported to be suitable reference genes in the skin of gilthead seabream as well [29].

Data are expressed as relative gene expression of each target gene (mean ± SEM). Statistical analysis (t-test) was performed using Statistical Package for the Social Sciences (SPSS) software v19.0. One or two asterisks denote significant differences when *p* < 0.05 or *p* < 0.01, respectively.

### ELISA assay

Total mucus IgM levels were analysed by ELISA as described elsewhere [70]. First, 100 μl per well of 1/5 diluted mucus were placed in flat-bottomed 96-well plates in triplicate and the protein coating was performed by overnight incubation at 4 °C with 200 μl carbonate–bicarbonate buffer (35 mM NaHCO_3_ and 15 mM Na_2_CO_3_, pH 9.6). After three rinses with phosphate buffered saline (PBS; Sigma-Aldrich) containing 0.05% Tween 20 (PBT, pH 7.3) the plates were blocked for 2 h at room temperature with blocking buffer containing 3% bovine serum albumin (BSA; Sigma-Aldrich) in PBT, followed by three rinses with PBT. The plates were then incubated for 1 h with 100 μl per well of mouse anti-gilthead seabream IgM monoclonal antibody (Aquatic Diagnostics Ltd.) (diluted 1/100 in blocking buffer), washed and incubated with secondary antibody anti–mouse IgG-HRP (diluted 1/1000 in blocking buffer; Sigma-Aldrich). After exhaustive rinsing with PBT, the plates were developed using 100 μl 0.42 mM 3,3,5,5-tetramethylbenzidine hydrochloride (Sigma-Aldrich) solution, freshly prepared in distilled water containing 0.01% H_2_O_2_ (Merck). The reaction was allowed to proceed for 10 min and stopped by the addition of 50 μl 2 M H_2_SO_4_ and the plates were read at 450 nm in a plate reader (BMG, Fluostar Omega). Negative controls were wells without mucus and wells without primary antibody, both in triplicates, whose OD values were subtracted for each sample value.

ELISA data were analysed by using t-test. Data are expressed as mean ± SEM. Statistical test was performed using SPSS software v19.0. Asterisks denote significant differences between groups when *p* < 0.05.

## Abbreviations

ACTB: Actin cytoplasmic
APOA1: Apolipoprotein a1
ARF: Actin-related protein
ARF3: ADP-ribosylation factor 3
ATP5B: Adenosine triphosphate synthase subunit beta
C3: Complement component 3
CAPG: Macrophage-capping protein
CS: Citrate synthase
ELISA: Enzyme-linked immunosorbent assay
GSN: Gelsolin
GST3: Glutathione s-transferase 3
H2A: Histone 2a
H2B: Histone 2b
H4: Histone 4
HSC70: Heat shock cognate 71 kDa protein
IEF: Isoelectric focusing
IgM: Immunoglobulin mu heavy chain
KRT: Keratin
NKEF: Natural killer enhancing factor
PEBP1: Phosphatidylethanolamine-binding protein 1
PRDX: Peroxiredoxin
SOD: Cu/Zn superoxide dismutase
TPI: Triosephosphate isomerase
YWHAZ: 14–3-3 protein beta/alpha-1
